# The impact of endometrial injury on reproductive outcomes: results of an updated meta‐analysis

**DOI:** 10.1002/rmb2.12348

**Published:** 2020-09-17

**Authors:** Chen Nahshon, Lena Sagi‐Dain, Martha Dirnfeld

**Affiliations:** ^1^ Reproductive Endocrinology and IVF Unit, Department of Obstetrics and Gynecology, Carmel Medical Center Ruth & Bruce Faculty of Medicine, Technion Haifa Israel

**Keywords:** endometrial biopsy, endometrial injury, endometrial scratching, repeated implantation failure, reproductive outcomes

## Abstract

**Background:**

It is still unclear whether endometrial injury (EI) has a beneficial effect on reproductive outcomes, and if so, the optimal procedure characteristics are not clear. All previous papers concluded that more research is needed, and as additional studies were recently published, the insights on EI have changed significantly.

**Methods:**

Searches were conducted in MEDLINE, Embase, Web of Science, and Cochrane Library, to identify randomized controlled trials examining the EI effect on IVF outcomes in women at least one previous failed cycle.

**Results:**

2015 references were identified through database searching. Ultimately, 17 studies were included, involving 3016 patients. Clinical pregnancy rate (CPR) (RR = 1.19, [95% CI 1.06–1.32], *P* = .003) and live birth rate (LBR) (RR = 1.18, [95%CI 1.04–1.34], *P* = .009) were significantly improved after EI. Number of previous failed cycles, maternal age, and hysteroscopy were found to be relevant confounders. Higher CPR and LBR were found when EI was performed twice, while performing EI once did not significantly improve reproductive rates.

**Conclusion:**

According to the present meta‐analysis, EI may be offered to younger patients with few previous failed cycles and should be additionally studied in an RCT comparing different timing and more than one EI before treatment.

## Graphical abstract

According to the present meta‐analysis, EI may be offered to younger patients with few previous failed cycles. As higher CPR and LBR were found when EI was performed twice, it should be additionally studied in an RCT comparing different timing and more than one EI before treatment.

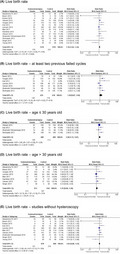

## 1 INTRODUCTION

Implantation success following in vitro fertilization (IVF) relays on several factors, including embryonic quality and endometrial receptivity.^1^, ^2^ Repeated implantation failure (RIF) after IVF and embryo transfer (ET) is a frequent problem many patients struggle with. Two definitions of RIF are acceptable in the academic and clinical fields. The recent definition refers to RIF as failure to achieve a pregnancy after transferring at least four good‐quality embryos in a minimum of three cycles in a woman under the age of 40 years.^3^ This annotation differs from the former definition that described RIF as failure to achieve pregnancy following two to six IVF cycles, with at least ten good‐quality embryos transferred.^4^

Endometrial injury (EI) was first described as a beneficial procedure for women with RIF during IVF treatments by Barash et al. in 2003.^5^ In this procedure, also known as endometrial scratching, the endometrium is locally intentionally damaged, usually by a Pipelle catheter.

Many studies have been published on the efficacy of EI and its true benefit on reproductive outcome, including several reviews and meta‐analyses, and basic science studies.^6^, ^7^ Our recently published meta‐analysis of randomized controlled trials (RCT) studied the EI effect in women with a least one previous failed IVF cycle.^8^ We showed that improved clinical pregnancy rates (CPR) and live birth rates (LBR) were apparent mainly in younger patients. However, in the subgroup of women with at least two previous failed cycles, the EI effect was not found beneficial.

Later, similar reviews were published.^9^, ^10^, ^11^ Vitagliano et al. showed improved reproductive outcomes in women with two or more previous failed cycles, with the greatest beneficial effect seen when double luteal EI was performed.^10^ Van Hoogenhuijze et al. found improved CPR but no improved LBR in women with at least two previous failed cycles, concluding that it is still unclear whether EI improves IVF outcomes.^9^ In line with this meta‐analysis, Gui et al. did not find any significant difference in CPR or LBR when including only RCT in their analysis.^11^

The recently published RCT by Lensen at al. concluded that EI did not improve LBR.^12^ Further published editorial recommending stated that it is “Time to Stop” offering EI to patients.^13^ However, in this RCT EI was performed in a time window that may have potentially skewed the results. The EI was performed between day three of the cycle preceding the IVF cycle and day three of the IVF cycle. However, in previous studies EI was mostly studied when performed during the preceding cycle. Moreover, two studies examining EI effect when performed during the same cycle presented harmful reproductive results.^14^, ^15^

Basic science studies proving the beneficial EI effect entailed two or more EI procedures,^5^, ^7^, ^16^, ^17^ thus raising the question whether it takes more than one EI to induce a proper immunological response. Optimal timing and quantity of EI have not yet been extensively discussed, yet they are potential confounders.

As all previous papers concluded that more research is needed, and due to accumulating new data on EI, we thought that an updated meta‐analysis is needed, emphasizing on analyzing the clinical outcomes when EI is performed more than once.

## 2 MATERIALS AND METHODS

This is an updated meta‐analysis, conducted according to the Preferred Reporting Items for Systematic reviews and Meta‐Analyses (PRISMA) statement,^18^ with search strategies, data extraction, and synthesis thoroughly described in our former paper.^8^

Study protocol, as previously described, is available at PROSPERO International prospective register of systematic reviews (registration number CRD42018092773). As no substantial changes were made, a new protocol was not required.^19^

Searches were conducted in the following databases: MEDLINE(R) by OvidSP interface and PUBMED, Embase, Web of Science and Cochrane Library, on January 28^th^, 2020

### 2.1 Study selection

Considered for inclusion were RCTs examining the EI effect on reproductive outcomes in women with at least one previous failed IVF cycle. In addition, we considered for inclusion studies that presented a subgroup analysis of patients with prior failed IVF attempts.

We contacted authors by email if insufficient information was published.

### 2.2 Outcomes measured

Our main outcomes were CPR and LBR. CPR was defined as the presence of a gestational sac presenting a positive heartbeat on transvaginal ultrasound. LBR was defined as the delivery of one or more live infants.

Secondary outcomes were multiple pregnancy and miscarriage rates. Multiple pregnancy rate was defined as the presence of more than one gestational sac on transvaginal ultrasound. Miscarriage rate was defined as fetal loss prior to the 20th week of gestation per clinical pregnancy.

Subgroup analyses were performed for known confounders such as at least two previous failed IVF cycles, maternal age, the use of hysteroscopy, and the number of times EI was performed before IVF treatment.

### 2.3 Assessment of risk of bias

Quality of RCTs was determined by the Cochrane Collaboration’s Risk of Bias tool Two independent reviewers made the assessment and if disagreements arose, the issues were resolved by discussion.

Publication bias was assessed by contour‐enhanced funnel plots, as well as the Begg and Mazumdar’s test and Egger regression asymmetry test. According to *Cochrane Handbook for Systematic Reviews of Interventions*, testing for publication bias by funnel plot asymmetry should not be conducted when less than ten studies are included in the meta‐analysis in order to avoid a false result. Thus, funnel plots were assessed only in comparisons including at least ten trials.

### 2.4 Data synthesis

RevMan 5.3 (Cochrane Collaboration, Oxford, UK) was applied for our quantitative synthesis. Heterogeneity across studies was assessed by the I‐squared statistic (an I‐squared statistic <25%—low level of heterogeneity, 25%–50%—moderate level, and >50%—high level). According to the heterogenicity, pooling of the results was performed using either the Mantel‐Haenszel fixed‐effects model or the Der Simonian‐Laird random‐effects model. The results were measured by risk ratio (RR), presenting the confidence interval (CI) and *P* value. A two‐tailed *P* < .05 was considered statistically significant. Sensitivity analyses were performed by omitting studies one‐by‐one from the analyses. Quality assessment was conducted according to the GRADE criteria.

## 3 RESULTS

### 3.1 Study selection

**FIGURE 1:**
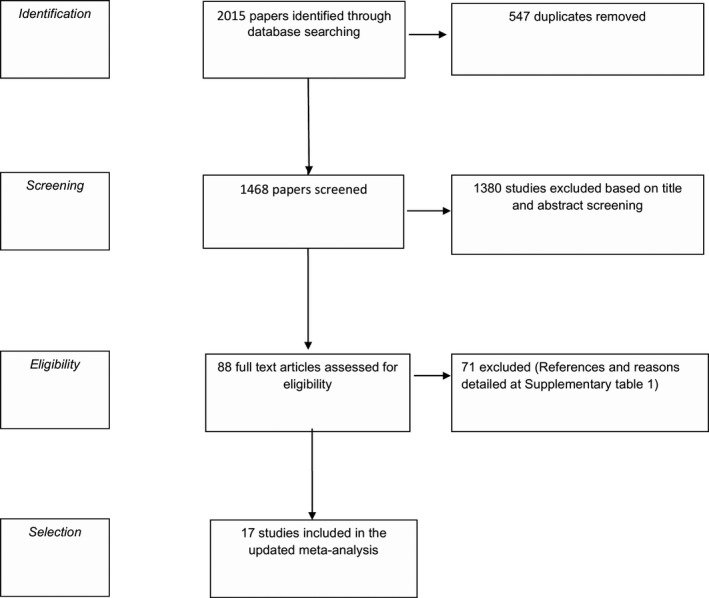
Flow diagram of database searching

Altogether, 2015 titles and abstracts were identified through database searching. All potentially relevant studies were reevaluated for inclusion. Figure 1 describes article handling. Supplementary Table S1 details the reasons for full‐text exclusion.

Finally, 17 references^12^, ^35^ comprising 3016 patients, 1498 in the EI group, and 1518 in the control group were included in our meta‐analysis.

All authors were contacted by email to provide additional information, only three responded.^12^, ^30^, ^33^

### 3.2 Study characteristics

Table 1 summarizes the characteristics of the included RCTs.

**TABLE 1:** Characteristics and outcomes of the included studies

First author (year)	Country (number of participants)	Inclusion Criteria	Average Age of patients included [years] Mean (SD)	Interventions	Outcomes measured	Notes
Aleyamma TK^20^	India (111)	Age <39	Intervention: 31.35 (4.20). Control: 32.02 (3.19)	Intervention group: Pipelle biopsy twice within 48 hours in the luteal phase prior to starting controlled ovarian hyperstimulation.	Clinical pregnancy, Live birth, Implantation, Multiple pregnancy, Miscarriage and Preterm delivery rates	
		At least one previous failed cycle		Control group: No endometrial biopsy.		
		Body mass index (BMI) <30 kg/m^2^				
		Follicle‐Stimulating Hormone level of <10 mIU/mL				
Baum^21^	Israel (36)	Age: 18‐41 years old	Intervention: 34.8 (4.3) Control: 34.4 (5.4)	Intervention group: Pipelle curette on days 9–12 and 21–24 of the menstrual cycle preceding IVF treatment.	Implantation, Live birth, and Clinical pregnancy rates	
		At least three previous failed cycles		Control group: Cervical Pipelle without biopsy		
		Good ovarian response in previous cycles				
Eskew^22^	USA (34)	Age: 18–43 years old	Not Available for subgroup analysis	Intervention group: Pipelle catheter biopsy performed once during the luteal phase in the cycle prior to embryo transfer.	Pregnancy test, Clinical pregnancy, Miscarriage, and Live birth rates	Subgroup analysis of patients with a prior failed cycle
				Control group: Sham biopsy, the Pipelle was not inserted into the cervix or uterus.		
Frantz^23^	France (52)	Age: 18–38 years old	Not Available for subgroup analysis	Intervention group: Pipelle biopsy once between day 20 to 24 of the preceding cycle.	Clinical pregnancy rates	Subgroup analysis of patients undergoing their second cycle.
		First or second IVF cycle.		Control group: Sham biopsy, the Pipelle was not inserted into the cervix or uterus.		
Gibreel^24^	Egypt (387)	Age <40 years old	Intervention: 30.2 (4.2) Control: 30.6 (3.9)	Intervention group: Pipelle biopsy on day 21 of the preceding IVF cycle, and then after two to three days.	Live birth, Clinical pregnancy, Miscarriage, and Multiple pregnancy rates	Intervention Group: 15 patients underwent hysteroscopy and scratching once. Control group: 12 patients underwent hysteroscopy once.
		At least one previous failed cycle		Control group: Cervical Pipelle without biopsy.		
Gurgan^25^	Turkey (305)	Age <40 years old	Intervention: 34.31 (3.83) Control: 33.64 (4.25)	Intervention group: endometrial injury on the 10th–12th day of the preceding cycle through office hysteroscopy.	Implantation, Clinical pregnancy, Twin pregnancies, Pregnancy losses, and Live birth rates	Endometrial injury was performed without energy modality during hysteroscopy.
		Repeated implantation failure		Control group: No endometrial injury		
Inal^26^	Turkey (100)	At least one previous failed cycle	Intervention: 29.6 (3.8) Control: 30.8 (4.5)	Intervention group: two consecutive endometrial biopsies with one‐week intervals during the luteal phase of the non‐transfer cycle.	Fertilization, Implantation, Clinical pregnancy, and Live birth rates	
		Good responders to hormonal stimulation		Control group: No endometrial biopsy.		
Karimzadeh^27^	Iran (115)	Age between 20‐40 years old	Intervention: 29.96 (3.93) Control: 29.73 (3.92)	Intervention group: Pipelle once on days 21–26.	Implantation and Clinical pregnancy rates	
		At least two previous failed cycles	Control group: No endometrial biopsy			
		No history of blood diseases				
Lensen^12^	New Zealand, United Kingdom, Sweden, Belgium, Australia (682)	Women planning IVF with their own oocytes	Intervention: 35.06 (4.06) Control: 35.26 (3.77)	Intervention group: Pipelle once between day 3 of the cycle preceding the IVF cycle and day 3 of the IVF cycle.	Live birth, Ongoing pregnancy, Clinical pregnancy, Ectopic pregnancy, Biochemical pregnancy, Miscarriage and Multiple pregnancy rates, stillbirth, pregnancy termination, pain, bleeding the day after the procedure, and maternal and neonatal outcomes.	Subgroup analysis of patients with a one and two prior failed cycles.
				Control group: No endometrial biopsy		Fresh or frozen embryo transfer.
Mak^28^	Hong Kong (229)	Patients undergoing natural cycle frozen embryo transfer	Intervention: 36.9 (3.32) Control: 36.78 (3.45)	Intervention group: pipette biopsy in the mid‐luteal phase of the preceding menstrual cycle.	Biochemical pregnancy, Implantation, Clinical pregnancy, Miscarriage, Multiple pregnancy, ongoing pregnancy, and live birth rates.	Frozen embryo transfer, Subgroup analysis of patients with a prior failed cycle
				Control group: endocervical manipulation.		
Narvekar^29^	India (100)	Age < 38	Intervention: 32.1 (3.4) Control: 32.3 (3.3)	Intervention group: Pipelle first on the day of hysteroscopy, and once again between 24th to 25th day of the non‐transfer cycle.	Clinical pregnancy, Live birth, Implantation, multiple pregnancy and miscarriage rates	All patients underwent hysteroscopy on day 7‐10 of the cycle prior to the embryo transfer cycle
		At least one previous failed cycle		Control group: No endometrial biopsy		
		Good responders				
Olesen^30^	Denmark (304)	Age: 18‐40 years old	Intervention: 31.9 (4.5) Control: 31.9 (4.6)	Intervention group: Pipelle in the luteal phase of the preceding cycle.	Clinical and ongoing pregnancy, Live birth, Implantation, Multiple pregnancy, and Miscarriage rates	
		Regular menstrual cycle (28‐32 days)		Control group: No endometrial biopsy		
		BMI 18‐32				
		IVF or ICSI patients				
		One or more prior implantation failures				
Pecorino^31^	Italy (80)	Age: 25‐37 years old	Intervention: 32 (NA) Control: 31 (NA)	Intervention group: Pipelle in the luteal phase of the preceding cycle.	Implantation and Clinical pregnancy rates	
		At least two previous failed cycles		Control group: Sham procedure using an embryo transfer catheter along the cervix inside the uterine cavity.		
		Normal thickness and endometrial ultrasound pattern				
		Good quality of seminal fluid of partner				
		Negative metabolic, genetic, and infective evaluation				
Shahrokh‐Tehraninejad^32^	Iran (120)	Age < 40 years old	Intervention: 29.5 (6.4) Control: 28.3 (5.6)	Intervention group: Pipelle biopsy on day 21 of their cycle before IVF.	Clinical Pregnancy, live birth, miscarriage, ectopic pregnancy, and Blighted ovum rates	Frozen embryo transfer
		At least two previous failed cycles		Control group: No endometrial biopsy		
		Normal uterus in hysterosalpingography (HSG), sonography, or hysteroscopy, and at least 7mm endometrium thickness at suppository progesterone administration day I				
Shohayeb^33^	Egypt and Saudi Arabia (210)	Age <39 years old	Intervention: 30.7 (4.5) Control: 30.6 (4.5)	Intervention group: hysteroscopy and endometrial scraping were done once in the follicular phase at day 4–7 in the cycle preceding the embryo transfer cycle.	Implantation, Clinical pregnancy, Live birth, and miscarriage rates.	All patients underwent hysteroscopy at day 4–7 in the cycle preceding the embryo transfer cycle.
		At least two previous failed cycles		Control group: Hysteroscopy only		
		Normal thin endometrium				
Singh^34^	India (60)	Age <35 years old	Intervention: 31.73 (2.5) Control: 32.10 (2.2)	Intervention group: endometrial scratching once between days 14 and 21 of menstrual cycle in the cycle prior to ET.	Implantation, Live birth, Ongoing pregnancy, Abortion and Miscarriage rates	
		At least one previous failed cycle		Control group: No endometrial biopsy		
		Good ovarian reserve				
		No uterine manipulation within the last 3 months				
Yeung^35^	Hong Kong (91)	Normal uterine cavity	Not Available for subgroup analysis	Intervention group: endometrial aspirate once on day 21 of in the preceding cycle.	Ongoing pregnancy, clinical pregnancy, implantation, live birth, multiple pregnancy, and miscarriage rates	Subgroup analysis of patients with a prior failed cycle
				Control group: No endometrial injury		

Five studies with appropriate subgroup analyses answering our inclusion criteria were included in our meta‐analysis.^12^, ^27^, ^29^, ^30^, ^35^ Due to high risk of bias in the randomization process and allocation according to the clinical case record number, the study published by Matsumato et al. was excluded from our analysis.^36^

Eleven studies included patients with at least one previous failed cycle,^12^, ^33^, ^35^ four studies included patients with at least two previous failed cycles,^23^, ^24^, ^25^, ^34^ and the remaining two studies^28^, ^32^ included patients with at least three previous failed IVF cycles (Table 1). Three studies, in addition to presenting the data for patients with at least one previous failed cycle, provided further data for patients with at least two previous failed cycles.^12^, ^22^, ^33^

The average age of patients in five studies was up to and including 30 years old,^24^, ^25^, ^31^, ^33^, ^34^ and above 30 in nine studies.^12^, ^20^, ^21^, ^22^, ^23^, ^26^, ^28^, ^32^, ^35^ Three studies did not report the average age of included patients with previous failed cycles as the included data originated from a subgroup analysis^27^, ^29^, ^30^ (Table 1).

Hysteroscopy was part of the protocol in four studies.^21^, ^25^, ^31^, ^32^ Gurgan et al.^32^ compared hysteroscopic endometrial injury versus no hysteroscopy in the control group. In the study published by Gibreel et al.^31^ 15 patients underwent hysteroscopy in the EI group, and 12 patients underwent hysteroscopy in the control group. In Narvekar et al.’s study,^21^ all patients underwent hysteroscopy, yet the EI was performed by a Pipelle catheter. Shohayeb et al.^25^ studied hysteroscopy with EI versus hysteroscopy alone.

As noted in Table 1, studies varied in inclusion criteria and in the EI procedure. Nine studies performed EI once on the luteal phase of the cycle preceding IVF treatment,^22^, ^23^, ^24^, ^26^, ^27^, ^29^, ^30^, ^34^, ^35^ two studies performed EI once on the follicular phase,^25^, ^32^ one study performed EI once between day 3 of the cycle preceding the IVF cycle and day 3 of the IVF cycle,^12^ three studies performed EI twice on the luteal phase^20^, ^31^, ^33^ and two studies performed EI twice, once in the follicular phase and once in the luteal phase.^21^, ^28^

### 3.3 Risk of bias of included studies

**FIGURE 2:**
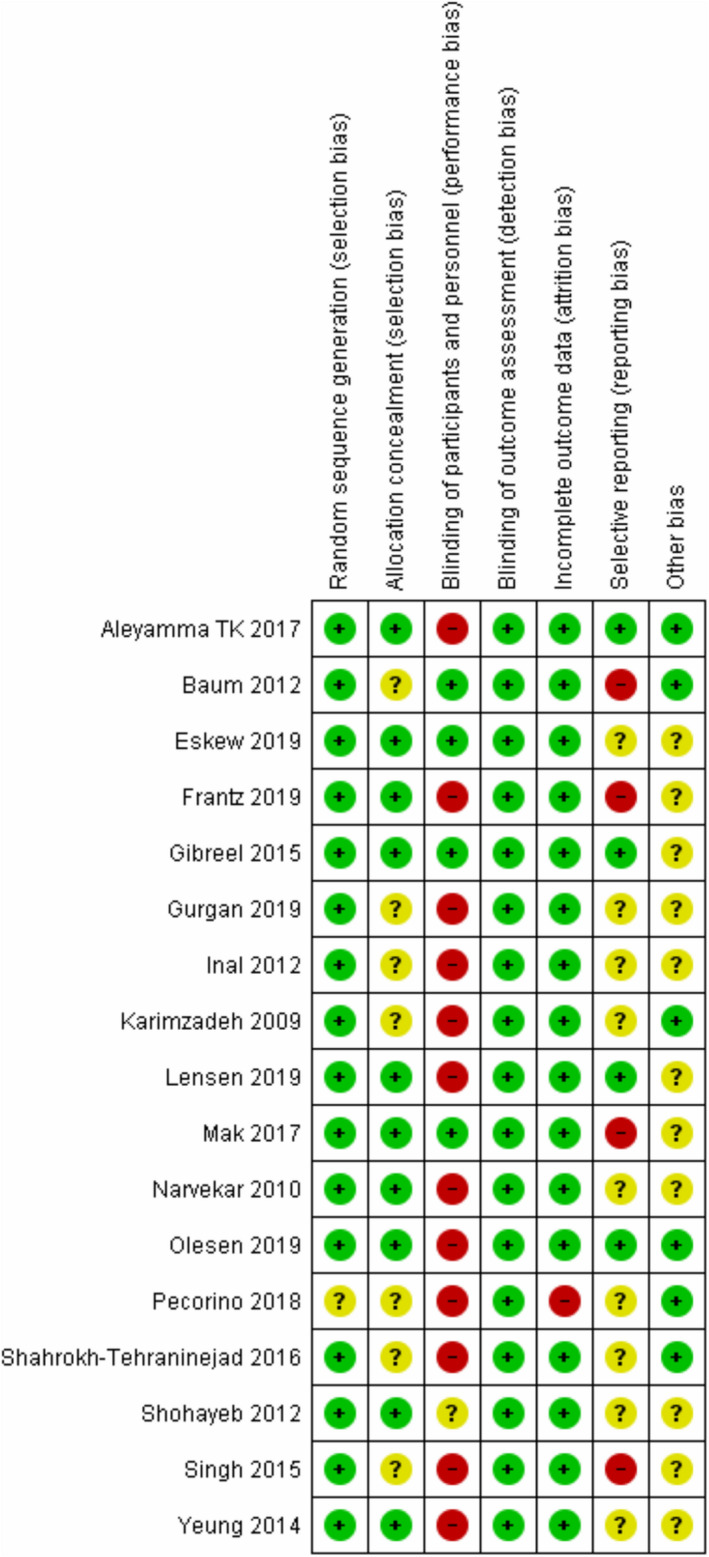
Risk of bias assessment

Figure 2 presents the risk of bias summary. One study had unclear risk of selection bias due to lack of description of the allocation sequence method,^23^ while seven studies had unclear risk of selection bias because allocation concealment method was not noted.^23^, ^24^, ^26^, ^28^, ^32^, ^33^, ^34^ Most studies were not blinded due to the nature of the procedure. However, we believe that lack of blinding was unlikely to affect the results, thus risk for detection bias was rated low for all studies. The risk for attrition bias was high in one study that did not present a CONSORT flow diagram or describe the follow‐up of patients.^23^ Reporting bias was rated high in four studies due to presentation of the results as percentage, presentation of ongoing pregnancies and LBR as one outcome or due to presentation of only one outcome in the subgroup analysis.^26^, ^28^, ^30^, ^35^ Unclear risk of reporting bias was also found in nine studies due to absent or retrospective clinical trial registration.^21^, ^23^, ^24^, ^25^, ^27^, ^29^, ^32^, ^33^, ^34^ Other factors of bias were unclear in six studies that involved antibiotics, steroids, or hysteroscopy in their treatment protocol.^21^, ^25^, ^26^, ^31^, ^32^, ^33^ Another five studies had unclear risk of bias due to the inclusion of a subgroup analysis.^12^, ^27^, ^29^, ^30^, ^35^

## 4 SYNTHESIS OF RESULTS

### 4.1 Clinical pregnancy rate

**FIGURE 3:**
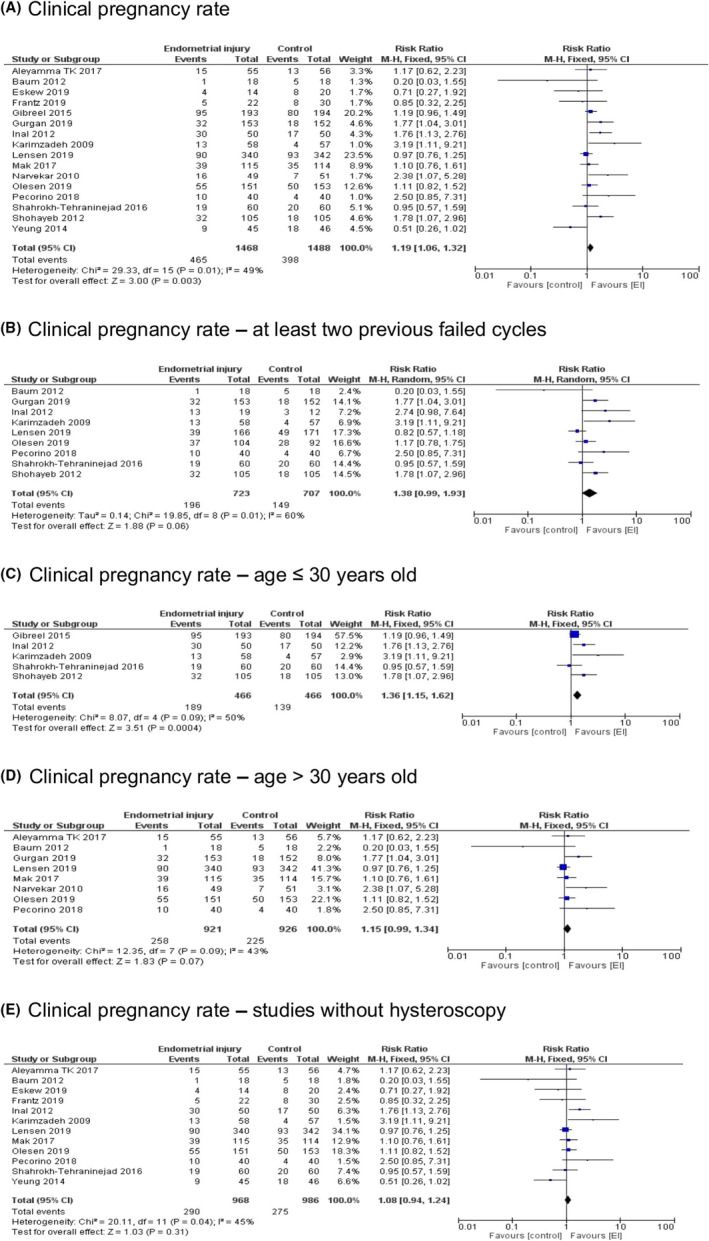
Clinical pregnancy rate—Forest plots. A, Clinical pregnancy rate, B, Clinical pregnancy rate—at least two previous failed cycles, C, Clinical pregnancy rate—age ≤ 30 years old, D, Clinical pregnancy rate—age > 30 years old, E, Clinical pregnancy rate—studies without hysteroscopy

CPR forest plots are presented in Figure 3. CPR was significantly higher in the EI group (RR = 1.19, [95%CI 1.06–1.32], *P* = .003). As Singh et al.^26^ provided ongoing pregnancy rates, they were not included in our CPR analysis.

Considering studies that included patients with at least two previous failed IVF cycles, CPR differences between the groups were of borderline significance (RR = 1.38, [95%CI 0.99–1.93], *P* = .06).

Subgroup analysis by maternal age showed that CPR was significantly higher in the EI group of patients with an average age up to and including 30 years old (RR = 1.36, [95%CI 1.15–1.62], *P* = .0004). However, in the group of patients with an average age above 30 years, CPR differences reached borderline significance (RR = 1.15, [95%CI 0.99–1.34], *P* = .07).

Analysis of studies that did not include hysteroscopy in the protocol resulted in an insignificant difference in CPR between the EI and control groups (RR = 1.08, [95%CI 0.94–1.24], *P* = .31).

**FIGURE 4:**
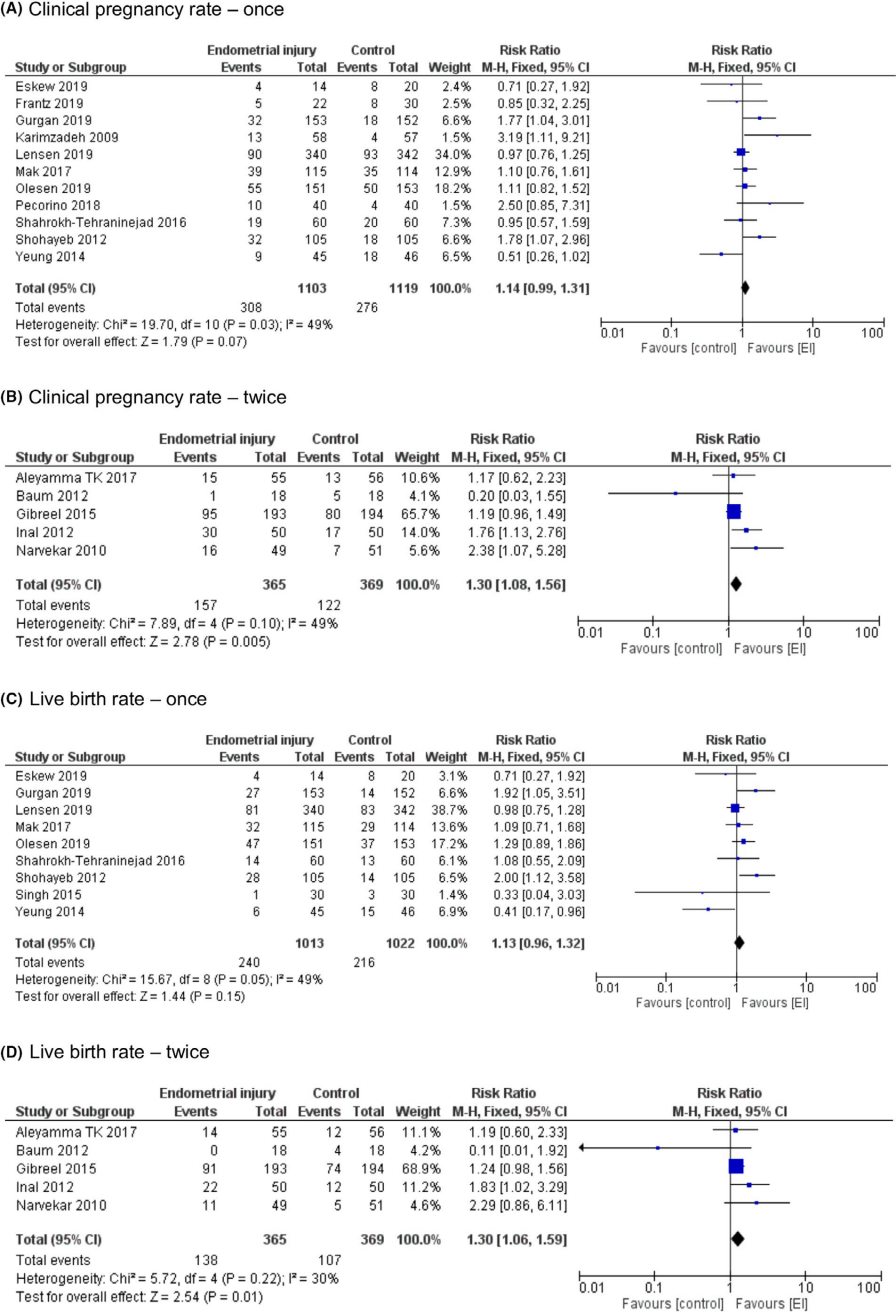
Subgroup analysis by the number of times of Endometrial injuries—Forest plots. A, Clinical pregnancy rate—once. B, Clinical pregnancy rate—twice. C, Live birth rate—once. D, Live birth rate—twice

Subgroup analysis by the number of times EI was performed showed that CPR differences between the groups were of borderline significance when EI was performed once (RR = 1.14, [95%CI 0.99–1.31], *P* = .07). While when EI was performed twice, significantly higher CPR was observed in the EI group (RR = 1.30, [95%CI 1.08–1.56], *P* = .005) (Figure 4).

### 4.2 Live birth rate

**FIGURE 5:**
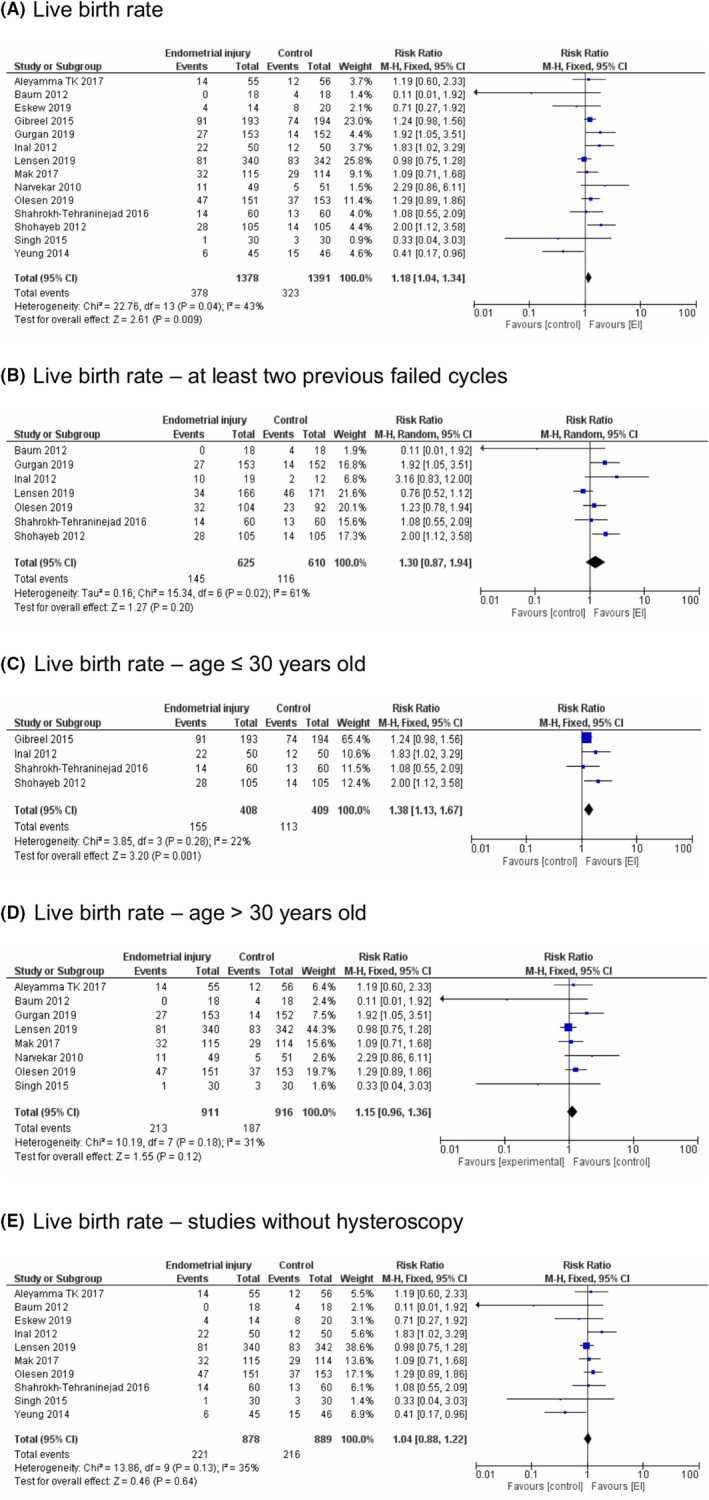
Live birth rate—Forest plots. A, Live birth rate. B, Live birth rate—at least two previous failed cycles. C, Live birth rate—age ≤ 30 years old. D, Live birth rate—age > 30 years old. E, Live birth rate—studies without hysteroscopy

LBR forest plots are presented in Figure 5. LBR was significantly higher in the EI group (RR = 1.18, [95%CI 1.04–1.34], *P* = .009).

Considering only studies that included patients with at least two previous failed IVF cycles, LBR did not differ between the EI and control groups (RR = 1.30, [95%CI 0.87–1.94], *P* = .20). Removal of the study published by Lensen et al. resulted in significantly higher LBR in the EI group (RR = 1.48, [95%CI 1.13–1.94], *P* = .004).

Subgroup analysis by maternal age showed similar results as in CPR. In the group of studies including patients with an average age up to and including 30 years old, the difference between groups was significant (RR = 1.38, [95%CI 1.13–1.67], *P* = .001). However, in the older group LBR did not differ between EI and control groups (RR = 1.15, [95%CI 0.96–1.36], *P* = .12).

In the analysis without the studies that included hysteroscopy, LBR did not differ between the EI and control groups (RR = 1.04, [95%CI 0.88–1.22], *P* = .64).

Subgroup analysis by the number of times EI was performed showed that LBR did not differ between the EI and control groups when EI was performed once (RR = 1.13, [95%CI 0.96–1.32], *P* = .15). However, when EI was performed twice, significantly higher LBR was observed in the EI group (RR = 1.30, [95%CI 1.06–1.59], *P* = .01) (Figure 4).

### 4.3 Miscarriage rate

Two studies reported miscarriage rate per positive pregnancy test or per cycle initiated (as opposed to per clinical pregnancy) and therefor were not included in this analysis.^22^, ^27^

**FIGURE 6:**
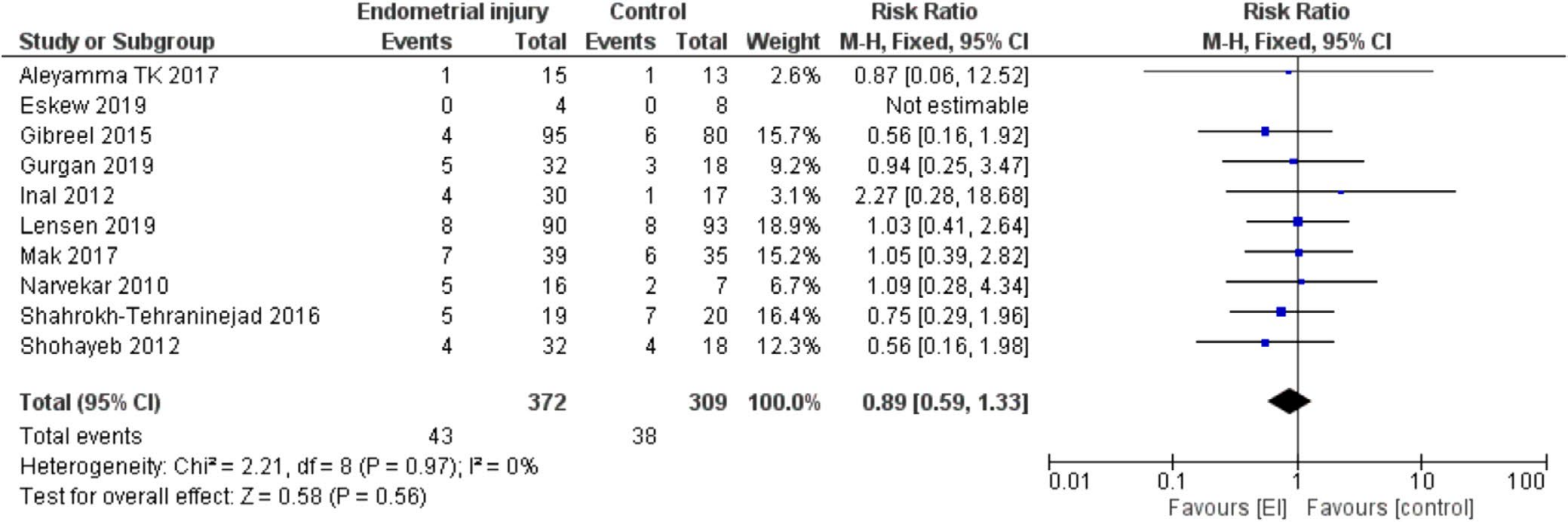
Miscarriage rate—Forest plot

Figure 6 presents the forest plot for miscarriage rate. The outcome did not differ between the EI and control groups (RR = 0.89, [95%CI 0.59–1.33], *P* = .56).

The same effect was observed when considering only studies that included patients with at least two previous failed IVF cycles (RR = 0.95, [95%CI 0.54–1.68], *P* = .86, 243 women, five trials).

Subgroup analysis by maternal age showed similar results. Both in the group of patients with an average age up to and including 30 years old (RR = 0.74, [95%CI 0.40–1.36], *P* = .33, 311 women, four trials) and in the group patients with an average age above 30 years (RR = 1.02, [95%CI 0.59–1.76], *P* = 0.94, 370 women, five trials), miscarriage rate did not differ between EI and control groups.

In the subgroup analysis excluding studies that included hysteroscopy, no significant EI effect was found on miscarriage rate (RR = 1.01, [95%CI 0.60–1.72], *P* = .96, 383 women, six trials).

Subgroup analysis by the number of times EI was performed showed that miscarriage rate did not differ between the EI and control groups when EI was performed once (RR = 0.88, [95%CI 0.55–1.41], *P* = .59, 408 women, six trials) or twice (RR = .90, [95%CI 0.41–1.98], *P* = .80, 273 women, four trials).

### 4.4 Multiple pregnancy rate

**FIGURE 7:**
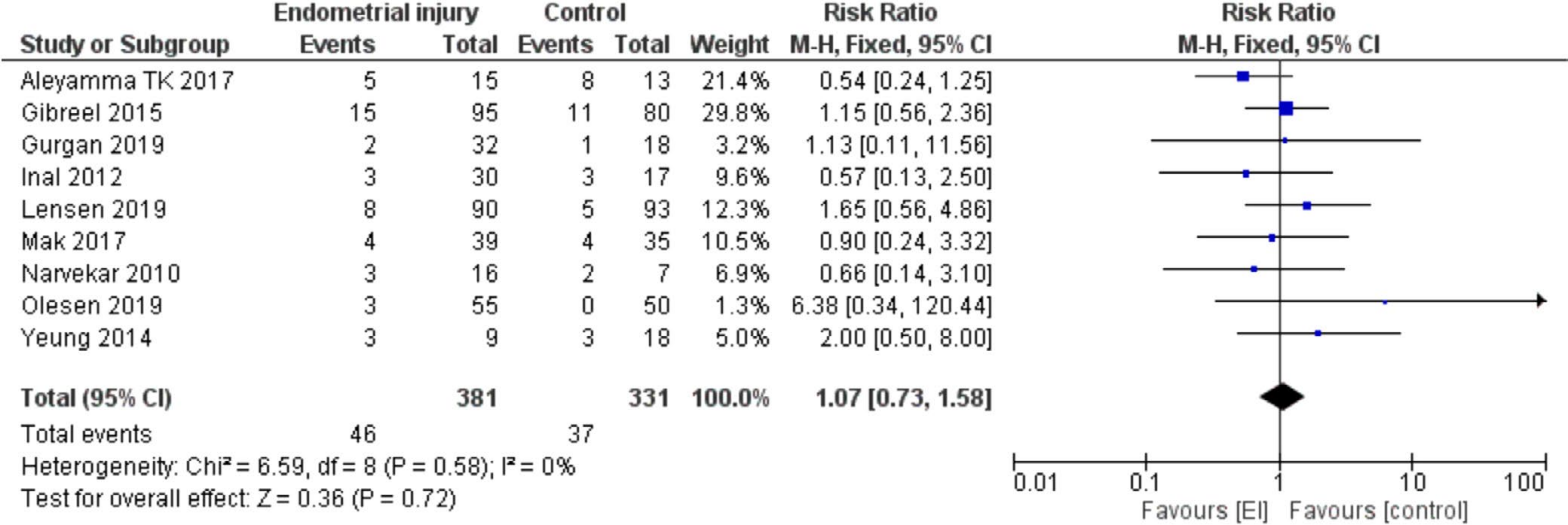
Multiple pregnancy rate—Forest plot

Figure 7 presents the forest plot for multiple pregnancy rate. The outcome did not significantly differ between the EI and control groups (RR = 1.07, [95%CI 0.73–1.58], *P* = 0.72).

Subgroup analyses were not conducted, as the number of studies per each comparison was low and not appropriate for a meta‐analysis.

### 4.5 Data synthesis

Most analyses resulted in low to moderate heterogenicity, with only two subgroup analyses (CPR and LBR of studies including patients with two or more previous failed cycles) presenting an I‐squared statistic of 60% and 61%.

Sensitivity analyses were conducted by omitting studies one‐by‐one from the analyses. In each comparison, this action did not change the significance of results, apart from one LBR subgroup of analysis, as mentioned above and in comparisons that resulted in borderline significance.

Comparisons of CPR, LBR, and miscarriage rates were assessed for publication bias by funnel plots (Supplementary Figures 1‐3). No asymmetry was detected; however, due to absent or retrospective clinical trial registration in nine studies (as mentioned), the risk for publication bias was defined as moderate.

Using the GRADE criteria, overall quality of existing evidence was initially described as “high” in light of RCTs regarding data acquisition. Nevertheless, the final grading was defined as “moderate,” mainly due to moderate risk of bias in most included studies and moderate inconsistency.

## 5 DISCUSSION

### 5.1 Main findings

Treatment of RIF patients is often frustrating, as the optimal management is not certain. The hope that an endometrial biopsy may help these couples has encouraged many physicians to examine it.^37^ The effect of EI on reproductive outcomes has been repeatedly studied; even since our recent meta‐analysis,^8^ many studies have been performed and published yielding mixed conclusions showing that the issue is still under a debate. The effect of more than one EI procedure has not been discussed in most studies. Our updated review adds new insights that may influence the clinical practice, emphasizing the possible need for more than one EI to achieve improved reproductive outcomes.

In the current meta‐analysis, we included only RCTs examining the yield of EI, in women with at least one previous failed IVF attempt. As presented in the results section, the EI had positive effect on CPR and LBR. Miscarriage and multiple pregnancy rates were not significantly affected by EI.

In patients with at least two previous failed cycles, CPR was improved with borderline significance, but LBR did not differ between groups. The EI effect on CPR and LBR was significant in the younger subgroup (≤30 years) and reproductive outcomes were significantly higher when EI was performed twice, as opposed to when performed only once.

The studies presented heterogeneity in inclusion criteria and patients characteristics; thus, we conducted subgroup analyses to identify potential confounders.

When analyzing only studies including patients with two or more previous failed cycles, the EI effect on CPR and LBR was no longer significant. However, worth mentioning is the higher heterogeneity in these comparisons. As presented above, only these comparisons presented an I‐squared statistic above 50%. In addition, due to borderline significance of the CPR subgroup comparison, a final conclusion is not evident. Noted is the study of Baum et al., which included patients with an average of 8.5 previous failed cycles, while most of the other studies included patients with less than three previous failed cycles.^28^ Baum et al. presented that the EI effect was not beneficial in their study population. All the above may imply that the relative contribution of infertility due to the endometrial factor decreases with any additional failed cycle and a high number of previous failed cycles may compromise the EI effect.

Subgroup analysis by maternal age yielded inconclusive results, as EI effect on CPR was significant in the younger subgroup, yet only marginally significant in the older subgroup. LBR rates were significantly higher after EI only in the younger subgroups. Worth mentioning, among all comparisons, the RR was highest in the younger subgroups (1.36 vs 1.15 in the CPR subgroup analysis and 1.38 vs 1.15 in the LBR subgroup analysis). These results suggest that age may have an impact on the success rates after performing EI. The endometrial factor may be potentially addressed by performing EI; however, it has been shown that the age‐related decline in female fertility is mostly related to oocyte quality rather than endometrial receptivity.^38^, ^39^

Hysteroscopy has been studied to have an independent EI effect, thus subgroup analyses omitting studies that included hysteroscopy as part of the treatment or protocol were conducted. These subgroup analyses showed that the CPR and LBR were no longer improved. Reaching a conclusion from these results is difficult as the studies varied in hysteroscopy use. This information emphasizes that hysteroscopy is indeed a confounding factor needs to be further addressed in future studies.

Our most interesting and surprising result refers to the optimal number of EI needed to be performed to achieve the best reproductive outcome. Studies included in our meta‐analysis performed EI once or twice, mostly in the luteal phase but not exclusively (Table 1). Subgroup analysis showed that CPR and LBR were significantly higher when EI was performed twice, as opposed to when performed only once. Moreover, the RR for CPR and LBR was higher in the comparisons including studies that performed EI twice (RR = 1.30 and RR = 1.30 vs. RR = 1.14 and RR = 1.13, respectively). Meaning, the magnitude of the EI effect was larger in these patients. Our results are in line with the meta‐analysis published by Vitagliano et al.^10^ showing that most optimal results were achieved after double luteal EI. In fact, basic science studies analyzing endometrial tissue entailed more than one biopsy, reaching up to four EI procedures.^5^, ^7^, ^16^, ^17^ These studies provide viable explanations to the mechanisms involved in improved implantation rates attributable to the inflammation process caused by EI. These studies demonstrated elevated pro‐inflammatory cytokines, upregulated endometrial gene expression, and increased macrophages and dendritic cells. Thus, integrating our results with those of basic science studies, it is reasonable to assume that one EI may just not be enough. In the first study, historically presenting the improved rates after EI, the procedure was performed four times.^5^ To our knowledge, no RCT has repeated this design. According to the most recent, IVF worldwide survey^40^ most clinicians around the world perform EI once in IVF cycles, being aware of recent publications on the topic, and mainly offering the procedure to RIF patients. Less than one percent of physicians perform EI more than three times. In view of the basic science effects proven and the results of the present meta‐analysis, an RCT studying the effect of performing EI multiple times in the cycle preceding IVF treatment is necessary.

### 5.2 Strengths and limitations

The present updated meta‐analysis presents the analysis of all published data from RCTs examining the effects of EI in women with previous failed IVF cycles. Also, we approached the authors of all studies for additional data to conduct more accurate comparisons. We present novel aspects of EI, regarding the optimal procedure characteristics and the possible need of more than one procedure for most favorable outcomes.

In view of varying inclusion criteria and EI application in the included RCTs, we were not able to eliminate all confounding factors (eg, stage and quality of embryos transferred). The type of EI may also have clinical impact as a Pipelle catheter, metal scratching, and aspiration may yield different results. In addition, of the 17 included studies, nine provided the reproductive outcomes in women with at least two previous failed cycles, more suitable for the definition of RIF. Methodological issues, also noted by Li et al.^41^ stress the need for future high‐quality RCTs, which in turn will translate into high‐quality evidence in reviews and meta‐analyses.

In our opinion, the optimal study that will prove whether an EI effect truly exists with minimal confounding factors is an RCT of EI in ovum donation cycles in RIF patients. Such study has not yet been published.

"/>

### 5.3 Conclusion

To conclude, the optimal population and procedure characteristics that may yield the greatest benefit from EI are still unknown and a matter of clinical discussion.^42^

Our data suggest that the relative contribution of endometrial receptivity to the chances of implantation may decrease with increased age and when performed in women with many failed cycles. The effect possibly increases when performed two or more times. Even though, we should embrace these results with caution, as sources of bias were detected in the analyzed studies.

In summary, EI should be offered restrictively, trying to identify which patient could truly benefit from the procedure. According to the present meta‐analysis, these may be the younger patients, with at least one IVF failure, and with EI performed twice in the cycle preceding the current treatment.

To confirm the observed beneficial effect of performing more than one endometrial biopsy, an RCT comparing EI in the follicular phase, luteal phase, and/or both should be conducted.

## CONFLICT OF INTEREST

Chen Nahshon, Lena Sagi‐Dain and Martha Dirnfeld declare that they have no conflict of interest.

## HUMAN/ANIMAL RIGHTS

This article does not contain any studies with human and animal subjects performed by the any of the authors.

## APPROVAL BY ETHICS COMMITTEE

Not applicable (systematic review and meta‐analysis).

## Supporting information

Fig S1

Fig S2

Fig S3

Table S1
