# Supplementary figures and images for: The impact of endometrial injury on reproductive outcomes: results of an updated meta‐analysis
Source: Reprod Med Biol. 2020 Sep 17;19(4):334–49. doi: 10.1002/rmb2.12348 (PMC7542009; doi:10.1002/rmb2.12348)

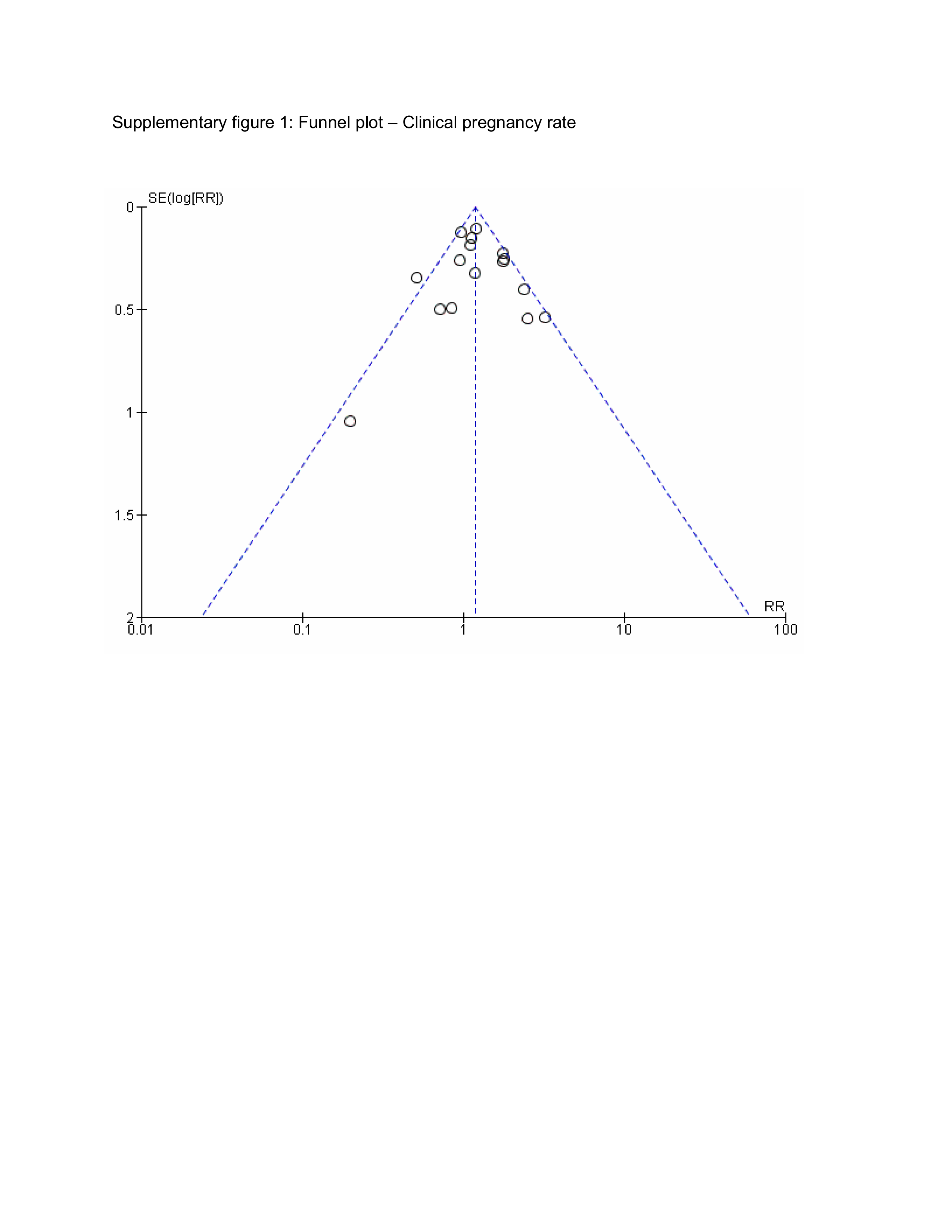

Supplement: Supplementary file 1 — Fig S1 [file RMB2-19-334-s001.tif]

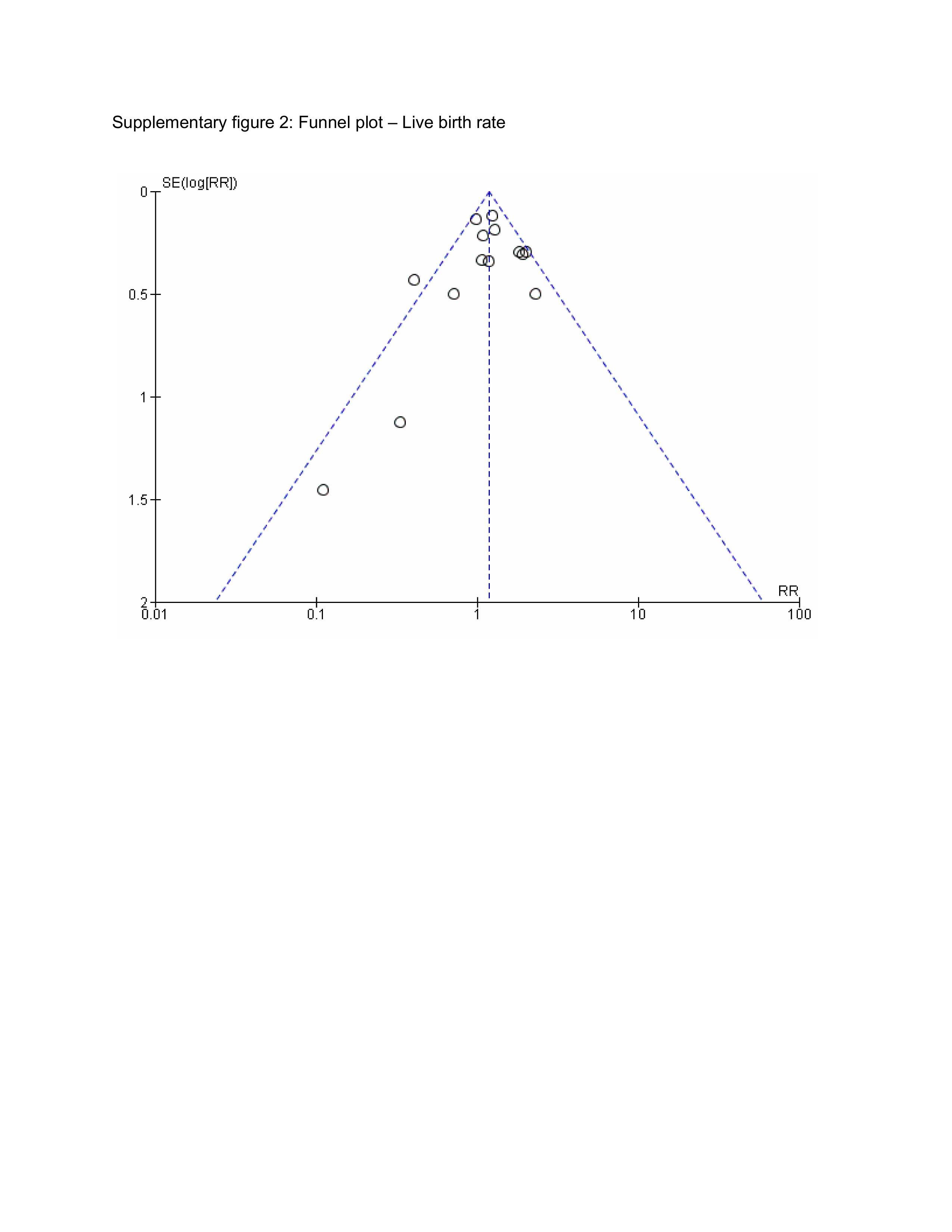

Supplement: Supplementary file 2 — Fig S2 [file RMB2-19-334-s002.tif]

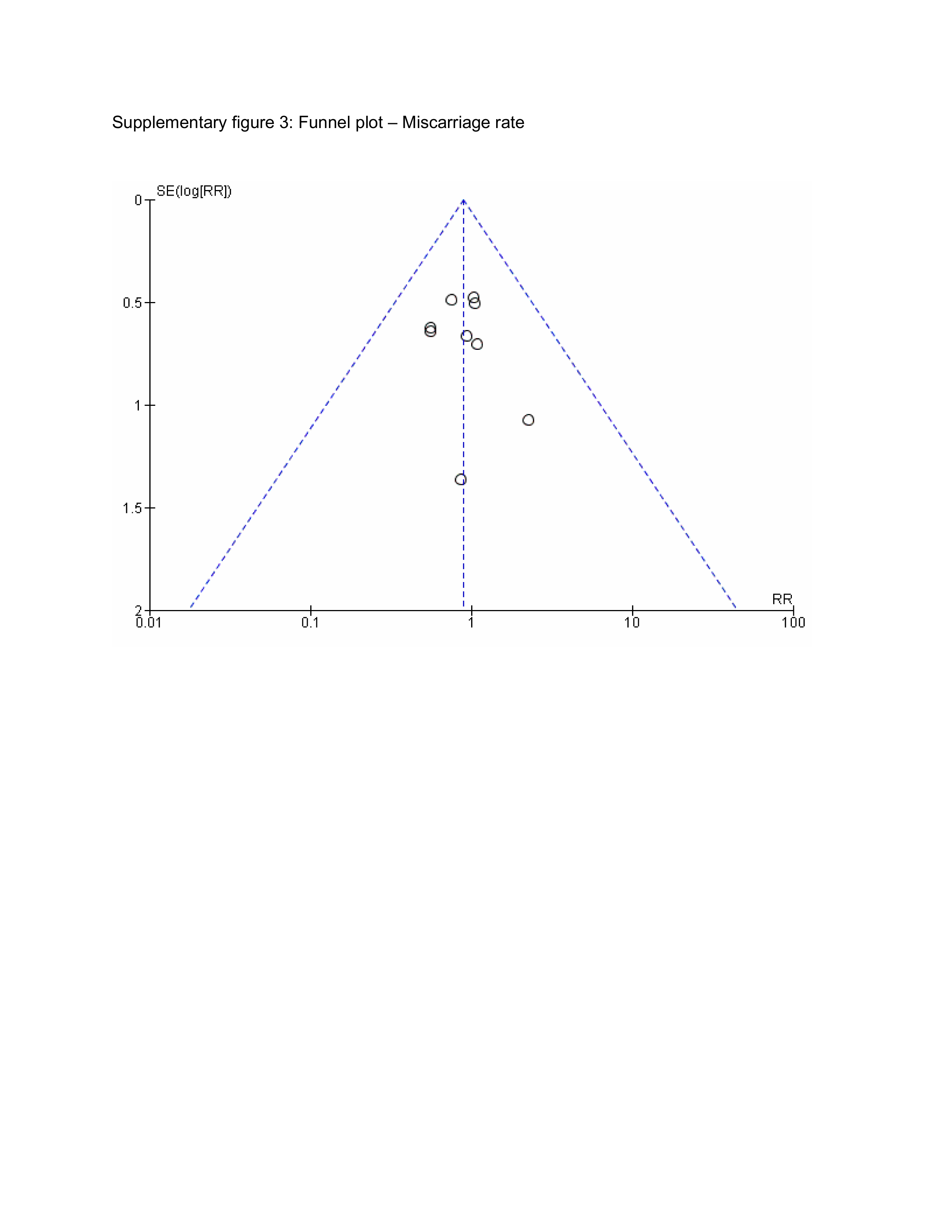

Supplement: Supplementary file 3 — Fig S3 [file RMB2-19-334-s003.tif]
