# Supplementary material for: The impact of endometrial injury on reproductive outcomes: results of an updated meta‐analysis
Source: Reprod Med Biol. 2020 Sep 17;19(4):334–49. doi: 10.1002/rmb2.12348 (PMC7542009; doi:10.1002/rmb2.12348)
Supplement: Supplementary file 4 — Table S1 [file RMB2-19-334-s004.docx]

Supplementary Table 1: Reasons for study exclusion at full-text evaluation stage

| **Reason for non-inclusion** | **References** |
| --- | --- |
| No previous failed cycles / unselected patients | ([1-17](#_ENREF_1)) |
| Prospective non-randomized studies | ([18-30](#_ENREF_16)) |
| Retrospective studies | (31-42) |
| Reviews | (43-60) |
| Abstracts, Editorials, Letters to the editor | ([61](#_ENREF_45)-68) |
| Study protocols | (69[-70](#_ENREF_50)) |
| Cross-sectional survey | (71) |

1. Aflatoonian A, Bagheri RB, Hosseinisadat R. The effect of endometrial injury on pregnancy rate in frozen-thawed embryo transfer: A randomized control trial. *Int J Reprod Biomed*. 2016;14(7):453-458. <http://www.embase.com/search/results?subaction=viewrecord&from=export&id=L611336168>.
2. Gibreel A, Badawy A, El-Refai W, El-Adawi N. Endometrial scratching to improve pregnancy rate in couples with unexplained subfertility: a randomized controlled trial. *J Obstet Gynaecol Res*. 2013;39(3):680-684. doi:https://dx.doi.org/10.1111/j.1447-0756.2012.02016.x
3. Hebeisha SA, Moiety FS, Samir M, Hussein M. Effect of endometrial injury on implantation and pregnancy rates: a randomised controlled trial. *Clin Exp Obstet Gynecol*. 2018;45(1):105-108.
4. Hilton J, Liu KE, Laskin CA, Havelock J. Effect of endometrial injury on in vitro fertilization pregnancy rates: a randomized, multicentre study. Arch Gynecol Obstet 2019; 299: 1159–64.
5. Karimzade MA, Oskouian H, Ahmadi S, Oskouian L. Local injury to the endometrium on the day of oocyte retrieval has a negative impact on implantation in assisted reproductive cycles: a randomized controlled trial. *Arch Gynecol Obstet*. 2010;281(3):499-503. doi:https://dx.doi.org/10.1007/s00404-009-1166-1
6. Liu W, Tal R, Chao H, Liu M, Liu Y. Effect of local endometrial injury in proliferative vs. luteal phase on IVF outcomes in unselected subfertile women undergoing in vitro fertilization. *Reprod Biol Endocrinol*. 2017;15(1):75. doi:https://dx.doi.org/10.1186/s12958-017-0296-8
7. Maged AM, Rashwan H, AbdelAziz S, et al. Randomized controlled trial of the effect of endometrial injury on implantation and clinical pregnancy rates during the first ICSI cycle. *Int J Gynecol Obstet*. 2018;140(2):211-216. doi:10.1002/ijgo.12355
8. Mahran A, Ibrahim M, Bahaa H. The effect of endometrial injury on first cycle IVF/ICSI outcome: A randomized controlled trial. *Int J Reprod Biomed*. 2016;14(3):193-198. <http://www.embase.com/search/results?subaction=viewrecord&from=export&id=L610950590>.
9. Mehrafza M, Asgharnia M, Abdollahiyan P, et al. The effects of local injury to the endometrium on pregnancy rate in ICSI: a randomized controlled trial. *Iran J Reprod Med*. 2010;Volume|:90. <http://cochranelibrary-wiley.com/o/cochrane/clcentral/articles/841/CN-01106841/frame.html>.
10. Nastri CO, Ferriani RA, Raine-Fenning N, Martins WP. Endometrial scratching performed in the non-transfer cycle and outcome of assisted reproduction: a randomized controlled trial. *Ultrasound Obstet Gynecol*. 2013;42(4):375-382. doi:https://dx.doi.org/10.1002/uog.12539
11. Polanski LT, Baumgarten MN, Richardson A, et al. Endometrial biopsy prior to assisted reproductive techniques (ART) does not improve treatment outcome in unselected patients. *Hum Reprod*. 2015;Volume|:i13. doi:10.1093/humrep/30.Supplement-1.1
12. Safdarian L, Movahedi S, Aleyasine A, Aghahosaini M, Fallah P, Rezaiian Z. Local injury to the endometrium does not improve the implantation rate in good responder patients undergoing in-vitro fertilization. *Iran J Reprod Med*. 2011;9(4):285-288.
13. Schulte M, Broughton DE, Eskew A, et al. Does endometrial mechanical stimulation (Scratch Test) improve pregnancy rates in in vitrofertilization cycles? a double blind randomized controlled trial. *Fertil sterility Conf 73rd Annu Congr Am Soc Reprod Med ASRM 2017 United states*. 2017;Volume|(3 Supplement 1):e366. <http://cochranelibrary-wiley.com/o/cochrane/clcentral/articles/078/CN-01417078/frame.html>.
14. Sherif A, Abou-Talib Y, Ibrahim M, Arafat R. The effect of day 6 endometrial injury of the ICSI cycle on pregnancy rate: A randomized controlled trial. *Middle East Fertil Soc J*. 2018;((Sherif A., ahmedsherif@med.asu.edu.eg; Abou-Talib Y.; Ibrahim M.) Department of Obstetrics and Gynecology, Ain Shams University, Egypt). doi:10.1016/j.mefs.2018.02.001
15. Shokeir T, Ebrahim M, El-Mogy H. Hysteroscopic-guided local endometrial injury does not improve natural cycle pregnancy rate in women with unexplained infertility: Randomized controlled trial. *J Obstet Gynaecol Res*. 2016;42(11):1553-1557. doi:https://dx.doi.org/10.1111/jog.13077
16. Zhao SY, Liu Y, Yu LP, Chao H, Liu MH, Zhao YY. Endometrial injury performed during the cycle preceding ovarian stimulation increases the biochemical pregnancy rate in unselected infertile women undergoing in vitro fertilization: A randomized placebo controlled trial. *Hum Reprod*. 2015;30((Zhao S.Y.; Liu Y.; Yu L.P.; Chao H.; Liu M.H.; Zhao Y.Y.) Beijing Obstetrics and Gynecology Hospital, Capital Medical University, Department of Reproductive Medicine, Beijing, China):i279. doi:10.1093/humrep/30.Supplement-1.1
17. Zhou L, Li R, Wang R, Huang H, Zhong K. Local injury to the endometrium in controlled ovarian hyperstimulation cycles improves implantation rates. *Fertil Steril*. 2008;89(5):1166-1176. <http://ovidsp.ovid.com/ovidweb.cgi?T=JS&PAGE=reference&D=med6&NEWS=N&AN=17681303>
18. Armine T, Manik G, Eduard H. Single and double endometrial scratching (ES) in infertile women with strict criteria of recurrent implantation failure (RIF). Gynecol Endocrinol 2019. DOI:10.1080/09513590.2019.1632085.
19. Barash A, Dekel N, Fieldust S, Segal I, Schechtman E, Granot I. Local injury to the endometrium doubles the incidence of successful pregnancies in patients undergoing in vitro fertilization. *Fertil Steril*. 2003;79(6):1317-1322. <http://ovidsp.ovid.com/ovidweb.cgi?T=JS&PAGE=reference&D=med4&NEWS=N&AN=12798877>
20. Chang E, Check JH, Liss JR, Choe J, Cohen R. An endometrial scratch can improve pregnancy rates in natural cycles of women with unexplained infertility given luteal phase support. *Fertil Steril*. 2017;108(3):e369. <http://www.embase.com/search/results?subaction=viewrecord&from=export&id=L618466558>.
21. Dunne C, Taylor B. Does endometrial injury improve implantation of frozen-thawed embryos? *Arch Gynecol Obstet*. 2014;290(3):575-579. doi:https://dx.doi.org/10.1007/s00404-014-3258-9
22. Farzadi L, Fakour A, Ghasemzadeh A, et al. The Effect of Local Endometrial Injury and GnRH Agonist on Pregnancy Rate in Patients With Recurrent Implantation Failure. *Int J Womens Heal Reprod Sci*. 2016;4(1):34-37.
23. Guven S, Kart C, Unsal MA, Yildirim O, Odaci E, Yulug E. Endometrial injury may increase the clinical pregnancy rate in normoresponders undergoing long agonist protocol ICSI cycles with single embryo transfer. *Eur J Obstet Gynecol Reprod Biol*. 2014;173(e4l, 0375672):58-62. doi:https://dx.doi.org/10.1016/j.ejogrb.2013.11.005
24. Huang SY, Wang C-J, Soong Y-K, et al. Site-specific endometrial injury improves implantation and pregnancy in patients with repeated implantation failures. *Reprod Biol Endocrinol*. 2011;9(101153627):140. doi:https://dx.doi.org/10.1186/1477-7827-9-140
25. Kumbak B, Sahin L, Ozkan S, Atilgan R. Impact of luteal phase hysteroscopy and concurrent endometrial biopsy on subsequent IVF cycle outcome. *Arch Gynecol Obstet*. 2014;290(2):369-374. doi:https://dx.doi.org/10.1007/s00404-014-3211-y
26. Matsumoto Y, Kokeguchi S, Shiotani M. Effects of endometrial injury on frozen-thawed blastocyst transfer in hormone replacement cycles. Reprod Med Biol 2017; 16: 196–9.
27. Olesen MS, Starnawska A, Agerholm I, Forman A, Overgaard MT, Nyegaard M. Does endometrial scratching change the biological age of the endometrium?-a paired prospective study. *Hum Reprod*. 2017;32((Olesen M.S.; Agerholm I.) Horsens Hospital, Fertility Clinic, Horsens, Denmark):i312. <http://www.embase.com/search/results?subaction=viewrecord&from=export&id=L617483989>
28. Raziel A, Schachter M, Strassburger D, Bern O, Ron-El R, Friedler S. Favorable influence of local injury to the endometrium in intracytoplasmic sperm injection patients with high-order implantation failure. *Fertil Steril*. 2007;87(1):198-201. <http://ovidsp.ovid.com/ovidweb.cgi?T=JS&PAGE=reference&D=med5&NEWS=N&AN=17197286>
29. Siristatidis C, Kreatsa M, Koutlaki N, Galazios G, Pergialiotis V, Papantoniou N. Endometrial injury for RIF patients undergoing IVF/ICSI: a prospective nonrandomized controlled trial. *Gynecol Endocrinol*. 2017;33(4):297-300. doi:https://dx.doi.org/10.1080/09513590.2016.1255325
30. Tiboni GM, Giampietro F, Gabriele E, Di Donato V, Impicciatore GG. Impact of a Single Endometrial Injury on Assisted Reproductive Technology Outcome A Preliminary Observational Study. *J Reprod Med*. 2011;56(11-12):504-506.
31. Cunningham T, Maguiness S, Lesny P. Is a mock embryo transfer as effective as a formal endometrial scratch in an unselected population? *Hum Fertil Conf Fertil 2015 Br Fertil Soc Assoc Clin Embryol Soc Reprod Fertil United kingdom*. 2015;Volume|(4) (no pagination). doi:10.3109/14647273.2015.1060045
32. Dain L, Ojha K, Bider D, et al. Effect of local endometrial injury on pregnancy outcomes in ovum donation cycles. *Fertil Steril*. 2014;102(4):1048-1054. doi:https://dx.doi.org/10.1016/j.fertnstert.2014.06.044
33. Faghih M, Deniz S, Neal M, Amin S, Hughes E, Karnis M. Impact of active cycle endometrial biopsy on implantation and pregnancy rates in fresh IVF cycles in patients with history of implantation failure. *Fertil Steril*. 2013;100(3):S293. doi:10.1016/j.fertnstert.2013.07.1036
34. Garg N, Harthshone G, Ghobara T, Rai J, Keay S, Agarwal R. Effect of endometrial injury on reproductive outcome in assisted reproduction: an observational study. *Hum Reprod Conf 32nd Annu Meet Eur Soc Hum Reprod Embryol Finl*. 2016;Volume|:i284. doi:10.1093/humrep/31.Supplement_1.1
35. Izquierdo A, Rayward J, Moschetta M, Calomarde Rees M, Gomez De Segura R, Lopez L. Does an endometrial scratch affect pregnancy rates in egg-donation treatment patients with or without previous implantation failures? *Fertil Steril*. 2016;106((Izquierdo A.; Rayward J.) Reproductive Medicine, Procrea Tec, Madrid, Spain):e339. <http://www.embase.com/search/results?subaction=viewrecord&from=export&id=L612867405>.
36. Kanazawa E, Nakashima A, Yonemoto K, et al. Injury to the endometrium prior to the frozen-thawed embryo transfer cycle improves pregnancy rates in patients with repeated implantation failure. *J Obstet Gynaecol Res*. 2017;43(1):128-134. doi:https://dx.doi.org/10.1111/jog.13182
37. Levin D, Hasson J, Cohen A, et al. The effect of endometrial injury on implantation and clinical pregnancy rates. *Gynecol Endocrinol*. 2017;33(10):779-782. doi:10.1080/09513590.2017.1318369
38. Migueles Pastor B, Zamorano B, Hebles M, Sanchez P, Sanchez F. Endometrial scratching in single implantation failure patients improves pregnancy rates in vitrified embryo transfer. *Hum Reprod*. 2014;29((Migueles Pastor B.; Hebles M.) Ginemed, Laboratory., Sevilla, Spain):i215. doi:10.1093/humrep/29.Supplement_1.1
39. Murtinger M, B WI, Canic T, Vanderzwalmen P, Schuff M. Clinical outcome after endometrial scratching (ES) in IVF-patients with a history of implantation failure. *Hum Reprod Conf 32nd Annu Meet Eur Soc Hum Reprod Embryol Finl*. 2016;Volume|:i267. doi:10.1093/humrep/31.Supplement_1.1
40. Seval MM, Sukur YE, Ozmen B, et al. Does adding endometrial scratching to diagnostic hysteroscopy improve pregnancy rates in women with recurrent in-vitro fertilization failure? *Gynecol Endocrinol*. 2016;32(12):957-960. <http://ovidsp.ovid.com/ovidweb.cgi?T=JS&PAGE=reference&D=med8&NEWS=N&AN=27258405>
41. Vlismas A, Christopoulos G, Barsoum-Derias E, Ramos-Luque M, Trew G, Lavery S. The impact of endometrial scratch on clinical pregnancy and live birth rate in IVF cycles. *Hum Fertil Conf Fertil 2015 Br Fertil Soc Assoc Clin Embryol Soc Reprod Fertil United kingdom*. 2015;Volume|(4) (no pagination). doi:10.3109/14647273.2015.1060045
42. Werner MD, Forman EJ, Hong KH, Franasiak JM, Bergh PA, Scott RT. Endometrial disruption does not improve implantation in patients who have failed the transfer of euploid blastocysts. *J Assist Reprod Genet*. 2015;32(4):557-562. doi:https://dx.doi.org/10.1007/s10815-015-0435-0
43. Coughlan C, Harrity C, Laird S, Li TC. Local endometrial injury: a treatment strategy to improve implantation rates: a systematic review and meta-analysis. *Hum Reprod*. 2015;Volume|:i13. doi:10.1093/humrep/30.Supplement-1.1
44. Coughlan C, Yuan X, Demirol A, Ledger W, Li TC. Factors affecting the outcome of “endometrial scratch” in women with recurrent implantation failure. *J Reprod Med*. 2014;59(1-2):39-43. <http://ovidsp.ovid.com/ovidweb.cgi?T=JS&PAGE=reference&D=med8&NEWS=N&AN=24597285>
45. Dekel N, Gnainsky Y, Granot I, Racicot K, Mor G. The Role of Inflammation for a Successful Implantation. *Am J Reprod Immunol*. 2014;72(2):141-147.
46. El-Toukhy T, Sunkara S, Khalaf Y. Local endometrial injury and IVF outcome: a systematic review and meta-analysis. *Reprod Biomed Online*. 2012;25(4):345-354. doi:https://dx.doi.org/10.1016/j.rbmo.2012.06.012
47. Gnainsky Y, Granot I, Aldo P, et al. Biopsy-induced inflammatory conditions improve endometrial receptivity: the mechanism of action. *Reproduction*. 2015;149(1):75-85.
48. Gnainsky Y, Granot I, Aldo PB, et al. Local injury of the endometrium induces an inflammatory response that promotes successful implantation. *Fertil Steril*. 2010;94(6):2030-2036. doi:https://dx.doi.org/10.1016/j.fertnstert.2010.02.022
49. Gui J, Xu W, Yang J, Feng L, Jia J. Impact of local endometrial injury on in vitro fertilization/intracytoplasmic sperm injection outcomes: A systematic review and meta-analysis. J Obstet Gynaecol Res 2019; 45: 57–68.
50. Gunther V, Alkatout I, Junkers W, Darsari-Mettler A, Maass N, von Otte S. Endometrial scratching. An up to date overview. *Gynakologische Endokrinol*. 2017;15(4):287-291.
51. Ko JKY, Ng EHY. Scratching and IVF: Any role? *Curr Opin Obstet Gynecol*. 2016;28(3):178-183. doi:10.1097/gco.0000000000000264
52. Nastri Carolina O, Lensen Sarah F, Gibreel A, et al. Endometrial injury in women undergoing assisted reproductive techniques. *Cochrane Database Syst Rev*. 2015;Volume|(3). doi:10.1002/14651858.CD009517.pub3
53. Panagiotopoulou N, Karavolos S, Choudhary M. Endometrial injury prior to assisted reproductive techniques for recurrent implantation failure: a systematic literature review. *Eur J Obstet Gynecol Reprod Biol*. 2015;193(e4l, 0375672):27-33. doi:https://dx.doi.org/10.1016/j.ejogrb.2015.06.026
54. Potdar N, Achana F, Gelbaya TA. Network meta-analysis of interventions used to improve clinical pregnancy rate in women with implantation failure. *Hum Reprod*. 2014;29((Potdar N.; Gelbaya T.A.) University Hospitals of Leicester, Leicester Fertility Centre, Leicester, United Kingdom):i78-i79. doi:10.1093/humrep/29.Supplement_1.1
55. Potdar N, Mason-Birks S, Elson CJ, Gelbaya TA, Nardo LG. Endometrial injury for recurrent implantation failure: how robust is the evidence? *Hum Reprod*. 2012;Volume|. doi:10.1093/humrep/27.s2.78
56. Potdar N, Gelbaya T, Nardo LG. Endometrial injury to overcome recurrent embryo implantation failure: a systematic review and meta-analysis. *Reprod Biomed Online*. 2012;25(6):561-571. doi:https://dx.doi.org/10.1016/j.rbmo.2012.08.005
57. Santamaria X, Katzorke N, Simon C. Endometrial “scratching”: what the data show. *Curr Opin Obstet Gynecol*. 2016;28(4):242-249. doi:https://dx.doi.org/10.1097/GCO.0000000000000279
58. Simon C, Bellver J. Scratching beneath “The Scratching Case”: systematic reviews and meta-analyses, the back door for evidence-based medicine. *Hum Reprod*. 2014;29(8):1618-1621.
59. van Hoogenhuijze NE, Kasius JC, Broekmans FJM, Bosteels J, Torrance HL. Endometrial scratching prior to IVF; does it help and for whom? A systematic review and meta-analysis. Hum Reprod Open 2019; 2019: hoy025.
60. Zygula A, Szymusik I, Grzechocinska B, Marianowski P, Wielgos M. Endometrial injury for women with previous in vitro fertilization failure - does it improve pregnancy rate? *Neuroendocrinol Lett*. 2016;37(6):419-426.
61. Bonavita M, Mattila M, Ferreira FP, et al. Local endometrium injury/healing increases embryo implantation and pregnancy rates of in vitro fertilization treatments. *Hum Reprod*. 2011;26((Bonavita M.; Mattila M.) Huntington Medicina Reprodutiva, Hospital e Maternidade Santa Joana, Embryology, São Paulo, Brazil):i205-i206. doi:10.1093/humrep/26.s1.80
62. Deepika K, Pranesh G, Vinaykumar M, et al. Follicular phase endometrial stimulation (FES) in the transfer cycle in RIF cases: a Randomized control trial. *Hum Reprod Conf 32nd Annu Meet Eur Soc Hum Reprod Embryol Finl*. 2016;Volume|:i266. doi:10.1093/humrep/31.Supplement_1.1
63. Karim Zadeh Meybodi M, Ayazi M, Tabibnejad N. Effect of endometrium local injury on pregnancy outcome in patients with IVF/ICSI. *Hum Reprod Eur Soc Hum Reprod Embryol ESHRE 24th Annu Meet barcelona, 6-9 july 2008*. 2008;Volume|:i126 Abstract No: P-311 Poster. <http://cochranelibrary-wiley.com/o/cochrane/clcentral/articles/093/CN-00670093/frame.html>.
64. Odendaal J, Quenby S. A Randomized Trial of Endometrial Scratching before In Vitro Fertilization. N Engl J Med 2019; 380: 1777.
65. Rigos I, Basios G, Salamalekis G, Vrantza T, Siristatidis C, Despotidi A. Combination of endometrial injury and freeze all strategy in women with repeated implantation failures: Recent data from a pilot study. Eur J Obstet Gynecol Reprod Biol 2019; 234: e127.
66. Salama K, Saad S, Assaf A, Morsy A. Endometrial injury for unexplained infertility: a randomised control study. Eur J Contracept Reprod Heal care Conf 15th Congr Eur Soc Contracept Reprod Heal Hungary 2018; 23: 127.
67. Vidal C, Giles J, Labarta E, et al. Intentional endometrial injury trying to improve clinical outcomes of an oocyte donation program in patients without RIF. Interym analysis of a randomized controlled trial. Fertil Steril 2019; 112: e164–5.
68. Youssef AM, Shawki HE. Role of endometrial trauma prior to icsi in unexplained recurrent implantation failure RCT. Int J Gynaecol Obstet 2018; 143: 811‐.
69. Hoogenhuijze NE, Torrance HL, Mol F, et al. Endometrial scratching in women with implantation failure after a first IVF/ICSI cycle; does it lead to a higher live birth rate? The SCRaTCH study: a randomized controlled trial (NTR 5342). *BMC Womens Health*. 2017;Volume|(1):47. doi:10.1186/s12905-017-0378-y
70. Lensen S, Martins W, Nastri C, Sadler L, Farquhar C. Pipelle for Pregnancy (PIP): study protocols for three randomised controlled trials. *Trials*. 2016;Volume|(1):216. doi:10.1186/s13063-016-1301-9
71. Lensen S, Sadler L, Farquhar C. Endometrial scratching for subfertility: everyone’s doing it. *Hum Reprod*. 2016;31(6):1241-1244. doi:https://dx.doi.org/10.1093/humrep/dew053
